# Editorial: Pharmaceutically targeting hypoxia in the breast cancer microenvironment: mechanistic and translational approaches

**DOI:** 10.3389/fonc.2023.1265523

**Published:** 2023-08-23

**Authors:** Manjari Singh, Manash K. Paul, Subhadeep Roy

**Affiliations:** ^1^ Department of Pharmaceutical Sciences, Assam Central University, Silchar, Assam, India; ^2^ Department of Microbiology, Kasturba Medical College, Manipal Academy of Higher Education, Manipal, Karnataka, India; ^3^ Division of Pulmonary and Critical Care Medicine, David Geffen School of Medicine, University of California Los Angeles, Los Angeles, CA, United States; ^4^ Department of Pharmacology and Toxicology, National Institute of Pharmaceutical Education and Research, Kolkata, West Bengal, India

**Keywords:** breast cancer, hypoxia, drug delivery, tumor microenvironment (TME), HIF-1α

Hypoxia and tumor microenvironment (TME) significantly influence breast cancer development, progression, immune response, angiogenesis, and metastasis. Advances in multi-omic techniques have significantly improved our understanding of the underlying mechanisms of hypoxia and the development of targeting strategies for treating breast cancer (1).

Mehraj et al. published the first research on this topic. Doxorubicin is effective against triple-negative breast cancer (TNBC), although the development of doxorubicin resistance is a significant challenge. They stated that some novel therapeutics can boost doxorubicin’s efficacy while reducing its toxicity. Better TNBC therapy combinations may result from combining doxorubicin treatment with promising novel compounds or repurposed drugs. Additionally, the combined therapy will reduce the dosage and toxicity of doxorubicin. Adapalene, a third-generation retinoid, has shown promise in treating certain cancers. They examined the anti-cancer properties of adapalenein on TNBC cells, its combinatorial efficacy with doxorubicin, and the mechanism of action. Adapalene and doxorubicin synergistically reduce TNBC cell growth, colony formation, and migration. Adapalene and doxorubicin increased reactive oxygen species, causing Erk1/2 hyperphosphorylation and caspase-dependent cell death. Adapalene is a potential anticancer drug used alone or combined with current TNBC treatments (Mehraj et al.).

Continuing with drug remittance, Yong et al. discussed HIF-1α in the context of multidrug-resistant breast cancer. In their evaluation, *de novo* or acquired resistance remains a clinical challenge. Hypoxia is one mechanism of drug resistance. HIF-1α controls cell responsiveness to hypoxia. HIF-1α promotes tumor cell proliferation, invasion, angiogenesis, anaerobic glycolysis, and multidrug resistance. Their study focused on drug-resistant breast cancer and HIF-1α-targeted treatment (Yong et al.).

Cheng et al. noted that fast proliferation and delayed angiogenesis cause intratumoral hypoxia in breast cancer. HIF, a transcription factor, mediates metabolic reprogramming, tumor angiogenesis, tumor cell proliferation and metastasis, gene instability, and other physiological and pathological processes in the hypoxic milieu. Hypoxia alters tumor cells’ innate and acquired immunity to support tumor growth and suppresses immunological function. Thus, tumor microenvironment hypoxia offers a prospective target for breast cancer treatment resistance and poor efficacy. They also discuss the hypoxic mechanisms of breast cancer medication resistance and the latest HIF inhibitor-targeted medicines (Cheng et al.).

According to Singh et al., solid hypoxic tumor cells ferments glucose into lactate *via* aerobic glycolysis, which accumulates in the TME. Cancer cells fail to utilize lactate, so they release it into the TME, thereby increasing extracellular lactate and microenvironmental acidity. The cancer microenvironment also absorbs lactate under different pathophysiological circumstances. Lactate vanishes immediately in the cancer microenvironment, a mystery. Recent discoveries have illuminated the significance of lactic acidosis in cancer microenvironment. Lactate suppresses immunity and initiates angiogenesis and invasiveness in cancer cells *via* the *de novo* fatty acid synthesis pathway. In tumors that are normoxic, moderately hypoxic, and severely hypoxic, lactate reprograms the lipid biosynthesis pathway to create a metabolic symbiosis. In oxygen scarcity, highly hypoxic cancer cells cannot synthesize polyunsaturated fatty acids (PUFA) and release lactate into the TME. Lactate from the TME is taken up by the normoxic tumor cells and transformed back to PUFAs after a series of processes to be used by severely hypoxic cancer cells. Lactate plays a significant role in various biological processes, although its precise molecular mechanism remains elusive. This review examines the role of lactate in angiogenesis, invasiveness, immune suppression, and lipid synthesis reprogramming (Singh et al.).

Thomas et al.‘s review takes a different method. Mutagenesis and cancer cell growth are known to occur in hypoxic microenvironments. The authors highlight the deliberate induction of localized hypoxia by the tumor cells to promote angiogenesis and production of growth factors that promote tumor growth and metastasis while promoting concurrent damage or mutagenesis of adjacent healthy tissue. Low oxygen levels reduce tumor-infiltrating lymphocyte (TIL) activation and recruitment, causing immunosuppression and immune surveillance. Hypoxic tumor endothelium suppresses the immune system in many ways, creating an immunosuppressive TME. Tumour endothelium anergy or non-responsiveness towards inflammatory signals precludes effector T cells from the TME. Tumour endothelium expresses endothelial-specific antigens and immunoinhibitory proteins such as Programmed death ligand 1, 2 (PDL-1, 2) and T cell immunoglobulin and mucin-domain containing-3 (TIM-3) to suppress T lymphocytes and promote regulatory T cells. The hypoxic microenvironment recruits immunosuppressive cells like the myeloid-derived suppressor cells (MDSCs), tumor-associated macrophages (TAMs), and Regulatory T cells (Tregs) in the TME. However, tumor blood vessels lack the organization of the normal tissue vasculature. Vascular normalization may improve tumor access in several tumor types and complement treatment. This paper briefly reviews immune-herbal therapy and immune-nutraceutical techniques that target tumor immunological evasion to boost immune response in the hypoxic TME. This study seeks to determine if these strategies can reduce breast cancer growth and prevent metastatic cell proliferation *via* new immunological switch points (Thomas et al.).

Luo et al. have demonstrated that breast cancer exhibits an upregulation and activation of HIFs due to persistent tumor hypoxia. Hypoxia-induced HIFs regulate glycolysis, angiogenesis, and metastasis, promoting breast cancer and poor prognosis by enhancing tumor invasion, metastasis, and drug resistance. Thus targeting the HIF pathway may enhance tumor targeting. This review analyzes the molecular mechanism associated with HIFs and the therapeutic strategies explicitly targeting HIFs in breast cancer. Drug delivery systems (DDSs) for targeting HIF are becoming more common due to the advances in nanotechnology and allied fields. They emphasized that HIF-targeted DDS may effectively target breast cancer, including DDS like liposomes, polymers, and metal- or carbon-based nanoparticles (Luo et al.).

In their study, Tang et al. provide an interesting take on the role of HIF1AN expression and breast cancer. HIF1AN reduces HIF-1α stability and transcription. Breast cancer patients with a decreased expression of HIF1AN exhibit reduced immunological infiltration and T-cell exhaustion and are correlated with an unfavorable prognosis. HIF1AN, clinical outcomes, and breast cancer immune involvement are not yet linked. Breast cancer cells expressed less HIF1AN than control specimens. HIF1AN expression serves as a predicted marker for breast cancer survival. In breast cancer, HIF1AN expression was linked to chemokines and immune cell infiltration, including neutrophils, macrophages, T helper cells, B cells, Tregs, monocytes, dendritic cells, and NK cells (Tang et al.).

Rastogi et al. evaluate NF-κB’s complementing involvement in the tumor microenvironment. NF-B helps tumor formation and maintenance, while HIF-1α aids cell proliferation and angiogenic signaling. PHD-2 may be the oxygen-dependent regulator of HIF-1α and NF-κB. Without oxygen and 2-oxoglutarate, the proteasome degrades HIF-1α. This mechanism activates NF-κB, unlike PHD-2-mediated IKK hydroxylation, which deactivates it. In hypoxic cells, proteasomes protect HIF-1α, activating transcription factors related to metastasis and angiogenesis. Hypoxic cells accumulate lactate due to the Pasteur Effect. MCT-1 and MCT-4 cells transport lactate from the blood to non-hypoxic cancer cells in the lactate shuttle. Lactate, converted to pyruvate, fuels oxidative phosphorylation in non-hypoxic cancer cells. OXOPHOS cancer cells switch from glucose-to-lactate-facilitated oxidative phosphorylation. OXOPHOS cells had PHD-2. NF-κB activation is unexplained. Pyruvate, a 2-oxo-glutarate inhibitor, accumulates in non-hypoxic cancer cells. Pyruvate-mediated competitive reduction of 2-oxo-glutarate in non-hypoxic cancer cells inactivates PHD-2. NF-κB canonically activates. 2-oxoglutarate inhibits PHD-2 in non-hypoxic cancer cells. FIH blocks HIF-1α from transcription. Through pyruvate-mediated competitive inhibition of PHD-2, NF-B controls cancer cell growth and proliferation (Rastogi et al.).

Srivastava et al. reviewed the role of different hypoxia-targeting approaches in overcoming TNBC resistance. Based on biological and clinical data, TNBC-related mortality is high worldwide. Hypoxia modulates TNBC’s glycolysis and angiogenesis pathways. Changes to these pathways promote cancer stem cell (CSC) enrichment and immune escape, which leads to tumor invasion, migration, and metastasis. Hypoxia also affects epigenetic plasticity and DNA damage response (DDR) to promote TNBC survival and progression. Hypoxia generates the low oxygen situation that alters HIF-1α signaling in the TME, allowing tumors to survive and resist treatment. Thus, suggesting the importance of target-based therapeutics to overcome TNBC’s resistance. Chemotherapy, radiotherapy, immunotherapy, anti-angiogenic therapy, adjuvant therapy, photodynamic therapy, adoptive cell therapy, combination therapies, antibody-drug conjugates, and cancer vaccines may target HIF-1α. While improving therapy options, they also discussed the intrinsic mechanism and HIF-1α targeting concerns. The authors further discussed the future and major hypoxia-induced signaling-targeted TNBC resistance treatments (Srivastava et al.).

This compendium of papers will help readers understand how tumor hypoxia and the environment prevent breast cancer and offer therapeutic options.

## Author contributions

MS: Writing – original draft, Writing – review & editing. MP: Writing – original draft, Writing – review & editing. SR: Conceptualization, Investigation, Resources, Supervision, Visualization, Writing – original draft, Writing – review & editing.
